# Fertility Preservation in Girls

**DOI:** 10.1155/2012/139193

**Published:** 2012-02-16

**Authors:** Jennia Michaeli, Michael Weintraub, Eitan Gross, Yehuda Ginosar, Vardit Ravitsky, Einat Eizenman, Eduardo Mitrani, Meital Lebovich, Neri Laufer, Stephen Kennedy, Ariel Revel

**Affiliations:** ^1^Department of Obstetrics and Gynecology, Hadassah-Hebrew University Medical Center, 91120 Jerusalem, Israel; ^2^Department of Pediatric Hematology-Oncology, Hadassah-Hebrew University Medical Center, 91120 Jerusalem, Israel; ^3^Department of Pediatric Surgery, Hadassah-Hebrew University Medical Center, 91120 Jerusalem, Israel; ^4^Department of Anesthesiology, Hadassah-Hebrew University Medical Center, 91120 Jerusalem, Israel; ^5^Bioethics Programs, Faculty of Medicine, University of Montreal, Montreal, QC, Canada H3C 3J7; ^6^Department of Cell and Developmental Biology, Institute of Life Sciences, The Hebrew University of Jerusalem, 91904 Jerusalem, Israel; ^7^Nuffield Department of Obstetrics and Gynaecology, John Radcliffe Hospital, University of Oxford, Oxford QX1 2JD, UK

## Abstract

Children that undergo treatment for cancer are at risk of suffering from subfertility or hormonal dysfunction due to the detrimental effects of radiotherapy and chemotherapeutic agents on the gonads. Cryopreservation of ovarian tissue prior to treatment offers the possibility of restoring gonadal function after resumption of therapy. Effective counseling and management of pediatric patients is crucial for preserving their future reproductive potential. The purpose of this article is to review recent literature and to revise recommendations we made in a 2007 article. Pediatric hemato-oncology, reproductive endocrinology, surgery, anesthesia and bioethics perspectives are discussed and integrated to propose guidelines for offering ovarian cryopreservation to premenarcheal girls with cancer.

## 1. Introduction

Ovarian cryopreservation has been offered to patients for more than a decade and, to date, more than 15 babies have been born worldwide after successful transplantation of ovarian tissue [1, 2]. The success of ovarian cryopreservation in adult women at risk for infertility secondary to exposure to chemotherapy and radiation therapy has led pediatric oncologists to consider ovarian tissue cryopreservation in prepubertal girls undergoing potentially gonadotoxic therapy. However, offering ovarian cryopreservation to young girls raises major medical, ethical, and legal issues unique to this age group that must be addressed. Firstly, the efficacy of the procedure in this setting is unclear, and it remains to be shown that ovarian tissue harvested from prepubertal girls can yield a successful pregnancy when retransplanted after puberty. Secondly, identifying the patients most likely to benefit from the procedure is complex, because of the difficulty in evaluating the risk for fertility impairment in young patients, especially when the time interval between administration of chemotherapy and clinical presentation of premature ovarian insufficiency is measured in years [3]. Lastly, since preservation procedures must be performed soon after diagnosis, while the patient herself is not old enough to provide informed consent, there are bioethical concerns regarding the validity of the process. The purpose of this article is to review recent literature and to revise our recommendations from the article published in 2007 [4].

## 2. Pediatric Hematology-Oncology Perspective

In 2010, a total of 10,700 children and adolescents under the age of 14 were diagnosed with cancer in the US alone. More than 80% will survive the disease [5]. For some common pediatric cancers such as Wilms' tumor, Hodgkin's disease (HD), and B-cell non-Hodgkin lymphoma (B-NHL) cure rates approach 90% [6]. These high cure rates result in a growing number of children who will become long-term cancer survivors [7]. However, survivors often face long-term sequelae of their treatment because of irreversible tissue damage including to the reproductive organs that may harm pubertal development. Alternatively, they may present in adulthood with infertility or premature menopause, defined as cessation of ovarian function below the age of 40 [3]. Clearly, removal of the reproductive organs during treatment of a malignancy will prevent patients from conceiving with their own gametes. However, chemotherapy and radiotherapy may also eliminate or severely compromise hormonal production, as well as a patient's reproductive potential, by damaging steroid-producing cells and gametes.

### 2.1. Effect of Chemotherapy on Reproductive Potential

Both female and male gonadal tissues are very sensitive to chemotherapy [8, 9]. The degree of damage is determined by the patient's sex and age, as well as the drug used and the dose administered [10, 11].

Some pediatric chemotherapy regimens such as MOPP (mustragen, oncovin, procarbazine, and prednisone) for HD, and high-dose cyclophosphamide and busulfan for bone marrow transplantation, cause sterility in a significant number of patients [12–16]. Other regimens such as high-dose cyclophosphamide for B-NHL and Ewing sarcoma are associated with a significant risk for fertility impairment. No comprehensive data exists on the exact rates of fertility impairment associated with current pediatric oncology therapeutic regimens.

All chemotherapeutic drugs affect gonadal function although some are considered more harmful than others [17]. For example, alkylating agents such as cyclophosphamide and procarbazine are categorised as high-risk drugs [8, 18], whereas vincristine and methotrexate are considered to have lower risks. The problem, however, when trying to advise patients and their parents is that most patients are treated with multiagent protocols, and the relative contribution of individual drugs is difficult to determine. Hence, it is virtually impossible to give the patient or his/her parents an accurate assessment of the risk to fertility, but rather the patients are considered to be at a low, intermediate, or high risk of infertility [19, 20].

### 2.2. Effect of Radiation on Reproductive Potential

Total-body irradiation (TBI) or radiation therapy delivered to the pelvis/abdomen may cause irreversible damage to the gonads. In both sexes, the degree of damage depends on the radiation dose and field, fractionation schedule, and the patient's age. In females, it has been shown that for a given dose of radiation, the younger the patient at the time of treatment, the later the onset of premature menopause is [21]. A radiation dose of 2 Gy is estimated to damage 50% of ovarian follicles irreversibly; doses ranging from 5 to 20 Gy cause complete loss of ovarian function resulting in sterility [22].

### 2.3. Reduction of Treatment-Related Gonadotoxicity

Several approaches have been developed to reduce treatment-related gonadotoxicity. The ideal approach is to design treatment regimens that will maintain high cure rates while decreasing or eliminating agents with significant tissue toxicity. However, despite a growing understanding of the biology of cancer, and the identification of molecular targets specific to the malignant cell, the concept of targeted therapy, affecting only the malignant clone and sparing normal tissues, is still, in clinical practice, the exception rather than the rule and most survivors of childhood cancer are still at risk for significant long-term side effects including effects on fertility.

Another approach to decreasing the risk to fertility is the use of gender-tailored therapy. Boys with HD, who are more susceptible to the sterilizing effects of chemotherapy, are treated with lower doses of chemotherapy combined with radiation. Conversely, girls, who have a prohibitively high risk of radiation-induced secondary malignancies (especially breast cancer) and are less prone to chemotherapy-induced gonadal damage, are treated with more intensive chemotherapy but without radiation when feasible [23, 24].

Reduced chemotherapy and/or radiation therapy in diseases with high cure rates such as HD is conceptually appealing and may be efficacious. This approach, however, is not feasible in many pediatric malignancies. For example, high-risk acute lymphoblastic leukemia, neuroblastoma, Ewing sarcoma, high-risk rhabdomyosarcoma, and high-grade B-cell lymphoma are all treated with dose-intensive schedules that rely heavily on the use of alkylating agents [25–28]. Treatment regimens for these cancers have not changed significantly in the last decade and most patients treated with these regimens are expected to become long-term survivors, and to experience significant gonadal damage. Female patients with stage III Wilms' tumor who receive whole-abdomen radiation therapy are also at a high risk for fertility impairment [29]. Unfortunately, it is unlikely that these regimens will be supplanted in the foreseeable future by equally effective but less gonadotoxic regimens. The same applies to pediatric patients undergoing bone marrow transplantation, the overwhelming majority of whom will develop gonadal failure [30]. Therefore, it is vitally important to develop effective approaches to fertility preservation that may be offered to preadolescent children who are about to receive cancer treatment that is associated with a high risk of gonadal damage.

## 3. Reproductive Endocrinologists' Perspective

In adult patients that have a partner, cryopreservation of embryos remains the most reliable method to preserve fertility [20]. Another efficient option is vitrification of mature unfertilized oocytes [31]. Both these methods require hormonal stimulation and delay of treatment, thus cannot be offered to young girls or patients that must receive urgent treatment. Cryopreservation of gonadal tissue can now be offered to patients with malignancies that require gonadotoxic therapy.

### 3.1. Indications for Ovarian Tissue Cryopreservation

As the extent of the insult to gonadal function varies across different diseases and treatment protocols, it is important to counsel patients effectively about their risk of infertility. A classification system has therefore been developed, which lists most malignant diseases and their associated treatments [32]. However, the precise course of any disease is never completely predictable despite the best attempts to estimate prognosis prior to treatment. For example, in a series of 58 girls <16 years old, Jadoul et al. showed that 14%, who initially received treatment that placed them at low- to-medium risk of premature ovarian failure, needed more aggressive gonadotoxic treatment in the months following cryopreservation [33].

Benign indications for fertility preservation procedures include haematological or autoimmune diseases, as well as certain genetic conditions such as Fragile-X and Turner syndrome which predispose women to premature ovarian failure. In addition, repeated surgery due to ovarian cysts or ovarian torsion may result in decreased ovarian reserve [33].

### 3.2. Ovarian Tissue Cryopreservation

The procedure of ovarian tissue cryopreservation consists of several steps following the harvesting of the tissue. After harvesting, ovarian tissue is promptly delivered to the laboratory. Aspiration of any follicles present should be performed before cryopreserving ovarian tissue. Immature oocytes obtained from premenarchal girls can be matured *in vitro* and cryopreserved for future fertilization [34]. Ovarian tissue is traditionally cryopreserved using a “slow freezing” method [35]. First, ovarian cortex is separated from the medulla. The cortex is then dissected into small fragments, to maximize permeation of cryoprotective agents into the cells; these must be used to protect the oocyte and surrounding stromal cells from freezing injuries [36]. Ovarian fragment size ranges between 5 mm [1] and 350 *μ*m in thickness [2]. The exact composition of the cryoprotective solution and the freezing protocol vary between institutions [37, 38]. Most commonly, the solutions contain permeating cryoprotectants such as dimethyl sulfoxide (DMSO), 1,2-propanediol, or ethylene glycol, in combination with nonpermeating substances such as sucrose and human serum albumin. Tubes containing immersed ovarian tissue fragments are gradually cooled by a programmable freezer that allows slow and stepwise decreases in temperature. When the temperature reaches −140°C, the tubes can be plunged into liquid nitrogen at −196°C for storage.

### 3.3. Experimental Techniques for Ovarian Tissue Storing

A recent and promising technique for storing ovarian tissue is “rapid freezing,” termed vitrification. Small ovarian cortical fragments are immersed for a short period in a highly concentrated cryoprotective solution. Without a slow cooling delay, the ovarian tissue is plunged directly into liquid nitrogen. This induces a glass-like state that avoids the formation of ice crystals, which may harm the oocyte and stromal cells. The efficiency and safety of the technique still need to be fully investigated before it becomes standard practice. It has been suggested that vitrification is superior to slow freezing in terms of follicular survival and tissue preservation in general [39, 40]. Others have reported that conventional freezing is a more suitable method for ovarian tissue cryopreservation than vitrification [41, 42]. Currently, new vitrification protocols are being developed and may achieve better results [43]. Cryopreservation of an intact ovary is challenging because cryoprotective agents cannot penetrate all cells equally. The vascular pedicle of the ovary must be harvested and carefully dissected, but it can be difficult to avoid damaging the ovarian vessels. The ovarian artery is usually perfused, via a catheter, with a heparinised physiological solution so as to drain all the blood from the ovary. Thereafter, the ovary is perfused by, and immersed in, a cryoprotective solution followed by a cooling process, using a slow freezing protocol, as described above [44].

### 3.4. Transplantation of Ovarian Tissue

Autotransplantation of ovarian tissue once the patient is well enough can take place in several anatomical sites: the normal ovarian (orthotopic site) or another (heterotopic site) location. Regardless of the site, transplantation of ovarian tissue fragments is performed without any vascular anastomosis as the tissue is sutured directly to the recipient site. Orthotopic transplantation is performed in or onto the remaining ovary or ovarian stump, or by transplanting the tissue into a peritoneal pocket created by the surgeon in the broad ligament or pelvic peritoneum of the ovarian fossa. Transplantation can be performed at laparoscopy [45] or laparotomy [46]. In the presence of intact and patent fallopian tubes, spontaneous conception has been reported after orthotopic transplantation [45]. Alternatively, oocytes can be aspirated from the transplanted tissue for IVF [47]. Successful transplantation of ovarian tissue has not yet been described in paediatric patients, mainly because the cryopreservation and transplantation techniques are relatively new. However, we can assume that significant numbers of patients will undergo transplantation in the near future given that harvesting and cryopreservation of ovarian tissue has been performed in children for more than a decade. In adults, 14 healthy babies have been born after autotransplantation of cryopreserved ovarian tissue (see review by [1, 2]). These results suggest that the harvesting, cryopreservation, storage, and transplantation procedures are both feasible and safe. Ovarian tissue in children is rich in primordial follicles that appear to survive the cryopreservation and transplantation insults well. Abir et al. showed that ovarian biopsy from a 5-year-old patient contained viable follicles after cryopreservation that were suitable for transplantation [48]. The tissue harvested from young children is therefore expected to yield positive results after transplantation. A work in mice that analysed ovarian function after transplantation of immature ovarian tissue demonstrated that immature ovarian grafting can restore spontaneous puberty and fertility [49]. All live births that have resulted from ovarian tissue transplantation have arisen from orthotopic sites. Heterotopic transplantation can be to any site in the body other than the ovary or the adjacent peritoneum, for example, the subcutaneous space of the forearm or the abdominal wall [50, 51]. Other sites that have been proposed include the uterus, rectus abdominal muscle, and the space between the breast and superficial fascia of the pectoralis muscle [52]. Clearly, spontaneous conception is impossible at such sites, and IVF treatment is required. The advantages of using heterotopic sites are that the transplantation procedure is easier and the oocytes are more accessible for aspiration during IVF treatment. However, no clinical pregnancies have been achieved from heterotopic sites even though ovarian function has been restored [51]. Whole ovary can be transplanted through microsurgical anastomosis of the ovarian vessels to a recipient site, either heterotopic or orthotopic. Although successful transplantation of a frozen-thawed whole ovary has not yet been described in humans, encouraging data have been published in sheep [53]. In humans, a successful pregnancy was achieved following a microsurgical anastomosis of an intact fresh ovary [54]. Several sites for transplantation have been proposed, including the deep inferior epigastric, and the deep circumflex iliac, pedicles [55]. The optimal site for transplantation remains to be determined in further studies.

### 3.5. Risks in Ovarian Tissue Transplantation

The biggest drawback to ovarian tissue transplantation, carried out without a vascular anastomosis, is that the graft may not survive. In the immediate period after transplantation there may be ischaemic damage to the tissue, resulting in massive follicular death; however, most primordial follicles survive this ischaemic insult [36, 56]. Surgical manipulations have been described to encourage prompt neovascularisation of the transplanted tissue, for example, a two-step procedure, involving the creation of granulation tissue one week before orthoptic transplantation, is believed to decrease the ischaemic damage [45]. Transplantation of a whole ovary with its vascular pedicle clearly avoids such an ischaemic period, as immediate reperfusion of the ovary should occur: in sheep, hormonal function is reported to have continued for 6 years following transplantation of whole ovaries [57]. The only other major risk of transplantation is the possibility of seeding malignant cells by reintroducing ovarian tissue containing micrometastases, as recently shown through quantitative reverse-transcribed polymerase chain reaction (RT-PCR) studies. In cryopreserved ovarian tissue from leukaemia patients, RT-PCR, and long-term xenotransplantation detected malignant cells, which had been missed histologically [58]. This risk is more evident in patients with hematological malignancies [59], but cannot be excluded in patients with solid tumors as well. For this reason, molecular studies are recommended prior to transplanting the tissue; long-term followup is also advisable to monitor for disease recurrence.

Success in molecular detection of malignant cells depends on the precise diagnosis and presence of genetic markers. In chronic myeloid leukemia (CML) the presence of BCR-ABL gene is characteristic and always allows for detection of leukemic cell contamination in ovarian tissue. In B- and T-cell lymphomas, as well as in acute leukemia (ALL), PCR for immunoglobulin and T-cell receptor gene rearrangements can be performed. Unfortunately, not all cases display a specific genetic marker that can be detected on PCR [59]. Histology and immunohistochemistry were unable to locate malignant cells within the cryopreserved ovarian tissue, and thus cannot be considered sufficient testing prior to transplantation [58, 60].

### 3.6. Function of Ovarian Tissue Graft

Ovarian activity usually returns approximately 4 months after transplantation [1]. This period corresponds to the time it takes for primordial follicles, which are the ones that principally survive freezing and the insult of transplantation, to mature into antral follicles. Ovarian activity is confirmed by tracking follicular development with ultrasound, detecting ovulation and measuring circulating sex hormones.

## 4. Pediatric Surgery Perspective

Ovarian cryopreservation involves the surgical harvesting of ovarian tissue. An important prerequisite for performing this procedure is a critical appraisal of the feasibility and safety of ovarian surgery in children. Pathological conditions of the ovary are encountered in infancy and childhood. Ovarian cysts, torsion, or masses, can be treated surgically in infancy and even the in neonatal period when indicated [61, 62].

### 4.1. Minimally Invasive Surgery

Over the last decade operative endoscopy and the concept of minimally invasive surgery has changed the practice of surgery. Following improvement and miniaturization of the required equipment, pediatric surgeons adopted laparoscopic and thoracoscopic surgery [63–65]. Laparoscopy has the advantage of exploring the abdominal cavity through a small incision, evaluating both ovaries before resection for fertility preservation. Laparoscopic oophorectomy is performed by isolating the fallopian tube from the ovary and gaining control of the ovarian blood supply. The ovary can be removed in a special bag through one of the trocar sites or a small lower abdominal incision. It is important to avoid using electrocoagulation on the ovarian surface to preserve the cortical tissue that contains follicles. The reported rate of complications is very low [66]. When performed by experienced surgical and anesthetic teams, oophorectomy for fertility preservation either by laparotomy or by laparoscopy, can be done with minimal complications.

The emerging technique of single port-laparoscopy requires a single umbilical incision rather than the standard 3-4 incisions and thus may improve cosmetic outcome [67, 68]. Due to the novelty of the technique, there is limited experience in children. Therefore, we believe that this surgical technique can be offered in selected cases [69].

### 4.2. Experience in Ovarian Tissue Cryopreservation in Children

We estimate that several thousands of women worldwide have undergone ovarian tissue cryopreservation. A recent publication from Denmark's registry, reports 18% of patients younger than 14 years of age [37]. Donnez and Dolmans reported successfully performed ovarian tissue cryopreservation in 59 girls under 16 years of age without complications [70]. In our program, about 15% of the patients who have undergone ovarian cryopreservation were under the age of 15 (see Table 1). We report no surgical complications in pediatric patients. None of the procedures had to be converted to open laparotomy. 

In the future, laparoscopic harvesting of the whole ovary including its vascular pedicle may be performed with the prospect of subsequent microvascular anastomoses of the ovarian vessels [44, 54]. It is important to take special care to resect the full length of the infundibulopelvic ligament, as it is crucial for the cryopreservation and transplantation procedures.

## 5. Pediatric Anesthesiology Perspective

Any anesthetic is associated with a risk of complications. Anesthetic complications may increase in the presence of additional risk factors due to either the subject's condition or the surgical procedure to be performed. These need to be offset against the benefit that is conferred upon the patient by the surgery or the risks to the patient of withholding surgery.

### 5.1. Benefit to the Patient

Although fertility preservation is not a life-saving procedure, fertility is in many patients' eyes the very essence of life and many patients are prepared to take significant risks in order to become pregnant. The discussion in this case is more complicated as the patients are children, the surgery is still experimental (albeit with extremely promising results) and fertility is not guaranteed. These factors will be discussed in greater depth in the next section; but if joint discussion between parents and the multidisciplinary medical team suggest that there is a genuine potential for fertility preservation, we feel that this justifies anesthetic risk in most cases. Sadly, this is usually one of many anaesthetics that these children will need and the offer of hope of fertility is an important component in their holistic care. Our primary focus in this section is on assessing anesthetic risk factors.

### 5.2. The Effect of Age on Anesthetic Risk

Anesthesia-related complications remain more common in pediatric patients than in adults. Currently, there are no clear guidelines regarding the appropriate age to harvest ovarian tissue. We previously suggested that harvesting should not be performed in girls under the age of 3 because of anaesthetic considerations [4]. However, Poirot et al. have reported a large series of paediatric patients who underwent ovarian tissue harvesting: 13 out of 47 were <3 years old, the youngest of them being 10 months old at the time of the surgery [71].

The majority of intraoperative anesthetic complications in children are respiratory complications, and intraoperative laryngospasm is among the most common and important of these. In a retrospective study of 130 cases of intraoperative laryngospasm over 10 years in one large institution, 57%, 68%, and 83% of laryngospasm were recorded in children younger than 3, 5, and 10 years old, respectively [72]. In a prospective study of 24165 anesthetics over a two year period, the incidence of adverse respiratory events per 1000 anesthetics increased with reducing age (<1 year: 36.1; 1–7 years: 15.3; 8–15 years: 8.6) [73]. A prospective study of 9297 pediatric anesthetics in a large single medical center also reported that older children were less likely to have laryngospasm and other perioperative respiratory adverse events (cough, desaturation, or airway obstruction) than were younger children [74]. However, there was no particular age cutoff that was associated with a step-wise change in anesthetic risk. Furthermore, that study showed only very modest effects of age on the risk for perioperative laryngospasm (RR 0.9; 95%CI 0.88–0.91; *P* < 0.0001); the relative risk decreased by 11% for each yearly increase in age. However, as the baseline probability for perioperative laryngospasm was only 2% (and only 8% in the highest risk patients), the impact on the incidence of perioperative laryngospasm is actually very small. The impact of age on perioperative desaturation (RR 0.95; 95%CI 0.94–0.96; *P* < 0.0001) was even smaller and there was no significant effect of age on perioperative bronchospasm (RR 0.99; 95%CI 0.96–1.02; *P* = 0.33). By contrast, the risk for perioperative laryngospasm increased markedly if a nonspecialist anaesthesiologist was managing the case (RR 3.85; 95%CI 2.47–5.98; *P* < 0.0001), if inhalation (rather than intravenous) induction was used (RR 2.38; 95%CI 1.79–3.17; *P* < 0.0001) and if there was anesthesia change-over during the case (RR 4.09; 95%CI 2.65–6.34; *P* < 0.0001).

From the balance of data currently available we feel that the 3-year-age cutoff is excessively conservative. Although this was appropriate when the procedure was more experimental, we would not now withhold anesthesia for laparoscopic oophorectomy for any child over the age of 1 year based solely on age without any other anesthetic risk factors. Below 1 year of age, each case should be assessed individually and should warrant careful multidisciplinary discussion, but here too, age alone should not be the overriding consideration.

### 5.3. Other Anesthetic Risk Factors

Operative laparoscopy in children [65, 75] involves the insufflation of carbon dioxide into the peritoneal cavity to a preset pressure, typically 13 cm H_2_O. This is associated with several physiological challenges, including impaired venous return, hypotension, impaired respiratory compliance and hypercarbia. The effects on respiratory compliance are accentuated by the usual steep head-down position. However, these effects are limited to the duration of gas insufflation, which is typically brief and which may be curtailed or applied intermittently if necessary.

Clearly this procedure is not offered in healthy children and a wide range of haematological and oncological diagnoses will be present, each with their own spectrum of infectious, metabolic, and respiratory complications. Where possible these all require optimization prior to surgery. In cases of marked respiratory compromise (including pneumonia, pleural effusions, tense ascites, or hepatosplenomegaly) the added physiological burden of operative laparoscopy may be hazardous.

### 5.4. Balancing Risk and Benefit

It is advisable to combine ovarian tissue harvesting with other imaging or surgical procedures that require anaesthesia, such as bone marrow aspiration, lumbar puncture, or central line insertion [4]. Nevertheless, laparoscopy imposes additional physiological challenges on the patient, and it is strongly advised to correct hematological, infectious, and metabolic derangements where possible prior to surgery and to optimize the child's respiratory and volume status. We would recommend considering withholding the procedure from children with markedly compromised respiratory function. Laparoscopic oophorectomy should be performed by a surgical team with appropriate training and equipment [76] and that includes an anesthesiologist with specialist training in pediatric anesthesia. 

## 6. Bioethics Perspective

Fertility preservation in young girls through ovarian-tissue cryopreservation is still considered experimental and requires a procedure that involves certain risks. At the same time, with a number of pregnancies and deliveries already obtained by this method, it does provide young girls with a real potential benefit. A bioethical analysis should, therefore, address the ethical obligation of clinicians to offer this alternative and to discuss it with their patients as well as the ethical prerogative of parents to make this decision on behalf of their daughter.

The ethical principles of beneficence and respect for patient autonomy are usually interpreted in the bioethics literature as entailing an obligation to disclose any medical information that is pertinent to the patient's ability to make informed decisions, particularly when such information is directly related to a potential benefit [77]. Applying these principles can be relatively uncomplicated regarding medical procedures that are already accepted as the standard of care. It becomes more complicated regarding procedures that are still considered by most to be experimental and when the risk-benefit ratio is unclear.

While ovarian-tissue cryopreservation is still considered experimental, it can be argued that even at present, clinicians have an ethical obligation to inform their patients of its existence and discuss it with them. First, in terms of risk-benefit analysis, the risks are small while the benefits are significant. The procedure does not require delaying cancer treatment and the risk of general anesthesia can be mitigated by performing it during another medically indicated anesthetic session. On the other hand, it offers a tremendous potential benefit considering how devastating infertility can be later in life. Second, the bioethics literature encourages clinicians to acknowledge the “specific informational needs” of patients [77]. Considering this is currently the only option to preserve fertility in prepubertal girls and considering the irreversibility of the situation, it can be argued that patients have a specific need to be informed.

Do parents (or other legal guardians) have an ethical prerogative to make a decision regarding ovarian-tissue cryopreservation on behalf of their daughter? Parents have an ethical obligation to make decisions that are in the best interest of their child and they lose their parental liberty if they do not. In this case, to evaluate their girl's best interest they need to consider her present interest in minimizing risk against her future interest in fertility preservation. Since the risk involved in the procedure is small, the crucial factor is the nature of the girl's interest in fertility preservation.

A child's right to fertility preservation has been acknowledged in the bioethics literature as a “right in trust,” a unique type of right that ought to be safeguarded until the child reaches adulthood but can be violated before the child is even in a position to exercise it [78]. Davis claims that “therefore the child now has the right not to be sterilized, so that she may exercise the right to have children in the future” [79]. Since parents are authorizing cancer treatment that may impact future fertility, it is clear that they have the ethical prerogative to consent to ovarian-tissue cryopreservation which is the only way of protecting this “right in trust.” When the patient has already reached an age at which it is possible to explain to her the procedure's purpose, it is of course preferable to obtain her own assent as well.

## 7. Synthesis and Summary

The majority of children diagnosed with cancer are expected to be cured and become long-term survivors. A substantial number of these survivors are expected to face impaired fertility secondary to the gonadotoxic effects of chemotherapy and radiation. Pretreatment fertility counselling and fertility preservation have great impact on quality of life of cancer survivors [80].

Cryopreservation of female gametes is considered efficacious using vitrification techniques [31]. However, this requires hormonal stimulation and transvaginal aspiration of oocytes—both are not applicable in young girls.

During the last years, most medical centres have opened programs that offer various fertility preservation strategies. The American Society of Clinical Oncology (ASCO) has established practice guidelines—however, these are not specific for children [81].

We currently recommend that all girls planned for radiotherapy or chemotherapy be referred for consultation by physicians specializing in fertility preservation. Fertility preservation procedures should be offered only after a detailed discussion and informed consent process with the parents, and, where age appropriate, with the child herself. The experimental nature of the procedure should be clearly stated along with presentation of potential benefits. A multidisciplinary team including pediatric oncologists, reproductive endocrinologists, pediatric surgeons, and anesthetists, as well as social workers, must work closely together to provide optimal counselling for patients and their parents. The decision on whether to perform oophorectomy or biopsies of ovarian tissue depend on the patient's age, treatment gonadotoxicity, and physician's preferences. The approach should be discussed with the patient/parents prior to surgery.

Although we previously [4] recommended a lower limit of age: 3 years old, we currently consider that ovary cryopreservation can be safely offered even to younger girls since the potential benefits are currently more evident and the risks of anesthesia appear not to be increased. To provide an additional margin of safety, we propose that if another necessary medical or surgical procedure is planned (e.g., insertion of an indwelling venous catheter, bone marrow aspiration, or harvest), then ovarian cryopreservation should be performed during the same anesthetic session.

A summary of our revised guidelines for offering ovarian cryopreservation to girls with cancer is presented in Table 2.

The field of fertility preservation is constantly evolving, as new experience is acquired and new lessons are learned. Expertise in performing ovarian tissue cryopreservation and transplantation is increasing rapidly. We believe that all considerations and guidelines should be reevaluated and modified according to new data, in order to provide the best benefit-to-risk ratio for the patients.

We strongly believe that there is an ethical obligation of clinicians to offer fertility preservation and to discuss fertility issues with cancer patients or their parents, in order to provide the opportunity for future parenthood.

## Figures and Tables

**Table 1:** Ovarian cryopreservation in pediatric patients, Hadassah Medical Center (1997–2011).

Patient	Age at cryo (years)	Diagnosis	No. oocytes retrieved	No. oocytes frozen
1	15	Ewing sarcoma	0	0
2	15	Thalassemia major	0	0
3	15	Osteosarcoma, cervical cancer	18	2
4	15	AML	5	0
5	14	Ewing sarcoma	16	6
6	14	Rhabdomyosarcoma	5	3
7	13	Hodgkin's disease	0	0
8	13	Non-Hodgkin's lymphoma	0	0
9	13	Osteosarcoma	0	0
10	13	Hodgkin's disease	2	2
11	13	Ewing sarcoma	9	5
12	13	Hodgkin's disease	13	5
13	12	Ewing sarcoma	23	9
14	10	Sarcoma	0	0
15	10	Osteosarcoma	17	8
16	9	Osteosarcoma	6	2
17	8	Ewing sarcoma	8	2
18	5	Wilms' tumor	7	1
19	3	Neuroblastoma	4	1
20	3	Rhabdomyosarcoma	0	0

**Table 2:** Revised guidelines for offering ovarian cryopreservation to premenarcheal girls with cancer.

The treating team will conduct a detailed discussion of the risks and potential benefits of ovarian cryopreservation with the parents/guardians, and, where age-appropriate, with the patient.	
Ovarian cryopreservation will be offered in cases where necessary medical treatments pose a high-risk of ovarian damage (e.g, bone marrow transplantation, whole-abdomen radiationtherapy, and alkylator-intensive chemotherapy).	

Ovarian cryopreservation will be offered to girls over 1 years of age. Below 1 year of age, each case should be assessed individually in a multidisciplinary discussion (may be modified with increasing experience).	

Ovarian cryopreservation should preferably be performed during a medically indicated anesthetic session (insertion of an indwelling venous catheter, bone marrow biopsy, and autologous bone marrow harvest).	

Medical centers providing pediatric oncology care should form multidisciplinary teams to offer this treatment.	
